# Preparation of High-Strength and Excellent Compatibility Fluorine/Silicone Rubber Composites under the Synergistic Effect of Fillers

**DOI:** 10.1021/acsomega.2c06489

**Published:** 2023-01-17

**Authors:** RongPeng Zhao, ZhiGang Yin, Wei Zou, Hu Yang, Jie Yan, WenJiang Zheng, Hui Li

**Affiliations:** † School of Chemical Engineering, Sichuan University of Science & Engineering, Zigong643000, China; ‡ Organic Fluorine Material Key Laboratory of Sichuan Province, Zigong643000, China; § Zhonghao Chenguang Chemical Research Institute, Zigong643201, China

## Abstract

Fluorine/silicone composite rubber is widely used as a sealing material in aerospace, missile, automotive, petroleum, and other industries, but the traditional process does not use synergistic fillers to strengthen the composite system. In this research, fumed SiO_2_ and black caron (N990) were used as synergistic fillers, fluorine/silicone composite rubber was prepared by mechanical mixing process, and three different fluorine rubber systems were used to find the best composite material. The mechanical properties, thermal properties, aging properties, moderate strength properties, and microstructure of the composites were evaluated. Studies have shown that mixing the two can produce a certain interface interaction and effectively improve the compatibility. The physical properties of the material tended to decrease during the increase in the added amount of silicone rubber (MVQ). The maximum tensile strength of the hybrid material can reach 15 MPa. The optimal mixing ratio is fluororubber/silicone rubber (FKM/MVQ) = 9/1. At this time, the mechanical properties of the composite material are in the best state, and SiO_2_ and black caron (N990) have a reinforcing effect, which can effectively improve the mechanical properties. After the composite was kept at 200 °C for 48 h, the tensile strength and elongation of the best sample A1 were 99.5 and 97.0%, respectively, showing excellent anti-aging properties. This work provides a method to fabricate high-strength fluorine/silicone composites using synergistic fillers that may be used in heat-medium-sealed environments.

## 1 Introduction

Rubber products are widely used in automobiles, construction, electrical appliances, medical equipment, and other fields.^1−4^ Rubber materials and rubber composites have always been the focus of research.^5−7^ Among other things, rubber materials in applications are subject to changing with environmental changes, such as high temperature, low temperature, acid–base, oily and organic media, and so forth, leading to changes in the properties of rubber materials and thus limiting their use.^8−10^ At present, the above problems can be effectively solved by combining two or more rubbers or other materials through physical and chemical actions.^11,12^ Previous studies have made significant progress in improving the properties of rubber composites.^13,14^ In rubber composites, one kind of rubber is used as the matrix material, and it is a common method for researchers to select different auxiliary materials for composites. The selection of synergistic materials involves the design and preparation of materials, which can be another rubber or filler.^14,15^

Due to the high C–F bond energy and the small atomic radius, there exists the strongest electronegativity of the fluorine atom and the strong electron-withdrawing effect. Fluorine rubber effectively shields and protects the C–C bond in the main chain, giving the fluorine rubber excellent high-temperature resistance, aging and stability, and so forth.^16,17^ Therefore, it can be widely used as a sealing material in modern missiles, rockets, automobiles, petroleum industry, aviation, aerospace, and other fields. However, fluororubber is difficult in processing, has a high cost, and has a low-temperature performance, which limits its application in the above-mentioned fields. Therefore, research on improving these problems is imminent.^18−20^ Silicone rubber is a material composed of silicon chains containing methyl groups and a small amount of vinyl groups.^21^ Its main features are excellent low-temperature performance, long-term use at −60 °C, and poor resistance to solvents and oily media.^22^ Today, researchers are using it as a potential material to improve the properties of fluoroelastomers.^23^ On one hand, it can improve the low-temperature and processing performance of fluororubber, and on the other hand, it can improve the medium durability of silicone rubber. This replaces the expensive fluorosilicone rubber, which not only effectively reduces the cost but also realizes the improvement of economic value.^24^ Previous studies have shown that fluoroelastomers and silicone rubbers are thermodynamically incompatible systems, but they are technically compatible. Even if there is great difficulty in working together, they can reduce phase separation through high-strength physical interactions.^25^ In recent years, research on fluororubber and silicone rubber has been actively carried out,^26−29^ and their composite materials have also appeared.^30,31^ Sipra Khanra^32^ added fluorosilicone rubber and modified silica filler to a combination of fluorosilicone rubber and silicone rubber in different fillers. It could be found that the two are compatible and the mixture can be used in a wide range of applications as a sealing system scope, including oil- and fuel-resistant O-rings and seals. Wu^33^ investigated various manufacturing conditions, vulcanization systems, and compounding ratios to optimize the co-vulcanization and compatibility of fluororubber and silicone rubber, as well as the tensile strength and thermal properties of vulcanizates. The research shows that the application temperature of the blend is higher than that of fluororubber, the blend also has a good compatibility, and meanwhile, the mechanical properties and thermal properties of the blend are further improved. Ghosh^34^ studied the processing rheology and phase behavior of fluororubber and silicone rubber during mixing, finding that low-viscosity silicone rubber can improve the vulcanization behavior during mixing. There are many studies on fluorosilicone composites in the existing literature, but the strength of the composites produced is low, and there are few studies on synergistic fillers. The hybrid filler can effectively improve the dispersion and crosslink density, and the wear resistance of the mixed composite material has been significantly improved.^35^ On one hand, hybrid fillers can enhance the comprehensive properties of rubber, especially the mechanical properties and tensile properties. On the other hand, hybrid fillers can effectively improve the compatibility between rubbers. Therefore, hybrid fillers have superiority and advancement.^36^ In this research, fillers and masterbatches were mixed separately. It is convenient for the base material to be effectively combined with the rubber, thereby providing more vulcanization sites for the blended rubber to promote vulcanization. Besides, it can increase the crosslinked network during mixing. This can effectively increase the two-phase interaction, endow the fluorine/silicon composite with high strength, and improve compatibility.

This paper selected three fluororubbers as the matrix material, one silicone rubber as the composite material, and carbon black N990 and fumed SiO_2_ as the fillers for the fluorosilicon composite. A series of fluorine/silicone composite rubbers were prepared by mechanical mixing process. By characterizing the mechanical properties, thermal properties, surface properties, and aging properties of the composites, the optimal fluorosilicone rubber material was determined, and the effects of rubber types and mixed fillers on the properties of the composites were analyzed. In addition, its feasibility has been proved. Fluorine/silicone composite rubber material prepared in this study has excellent mechanical properties, good compatibility, and superior resistance to media. It is expected to be further applied in high-temperature medium oil seals, high-strength seals, and other systems.

## 2 Experiment

### 2.1 Materials

Fluorine rubber (FKM) [G203, Mooney viscosity 30 ± 5 (ML (1 + 10) 121 °C), composed of vinylidene fluoride, fluorine-containing olefin, and vulcanization point monomer], FKM [G503, Mooney viscosity 30 ± 5 (ML (1 + 10) 121 °C), composed of vinylidene fluoride, tetrafluoroethylene copolymerized with perfluoropropylene, and vulcanization point monomer], and FKM [G506, Mooney viscosity 45 (ML (1 + 10) 121 °C), copolymerized by vinylidene fluoride, tetrafluoroethylene, and perfluoropropylene and vulcanization point monomer] were purchased from Zhonghao Chenguang Chemical Research Institute Co., Ltd. Silicone rubber (MVQ) is methyl vinyl silicone rubber (110-1S, vinyl content 0.07–0.12 mol %, molecular weight 600,000) purchased from Dongjue Silicone Group Co., Ltd. Nanosilica (CQ-300) was provided by Emeishan Changqing New Materials Co., Ltd. 2,5-Dimethyl-2,5-di-*tert*-butyl hexane peroxide (DBPMH), triallyl isocyanurate (TAIC), carbon black (N990), and zinc oxide were purchased from Shanghai Qiaodi Chemical Co., Ltd. The following figure is the molecular formula structure of fluorine rubber.

### 2.2 Preparation of FMC Composites

FMC composites are produced by blending process. This particular experiment consists mainly of three simple steps. In the first step, fluororubber (FKM) (e.g., 90, 80, 70 g) and silica carbon black mixed filler (e.g., silica 4, 8, 12 g, carbon black 28, 24, 20 g) are mixed with a mixer (ZG-0.2KH) for 5 min. The fluororubber compound is transferred into an open mixer (ZG-160L), adjusting the nip to 1 mm. Then, TAIC (e.g., 3.15, 2.8, 2.45 g) and vulcanizing agent DBPMH (1.45, 1.40, 1.35 g) are added, and the materials are turned around to be mixed evenly, which thinly pass five times. The triangle bag is beaten more than five times and parked for 4 h after discharge. In the second step, silicone rubber (MVQ) (e.g., 10, 20, 30 g), zinc oxide (e.g., 4.5, 4.0, 3.5 g), and hydroxy silicone oil (e.g., 0.2, 0.4, 0.6 g) were mixed; then vulcanizing agent DBPMH (e.g., 1.45, 1.40, 1.35 g) was added after mixing in the open mill for 5 min, adjusting the roll distance to 2 mm, and the sheet was left for 4 h for standby. The third step is to add the fluororubber mixture obtained in the first step to the open mill to adjust the roll distance to less than 1 mm and wrap the rolls, then add the silicone rubber mixture obtained in the second step five times, and turn the materials left and right to be mixed. After refining evenly, the materials are thinly passed five times, the triangle bag is beaten more than five times, and the tablets are left for 4 h for later use. Next, the formed blend was subjected to one-stage molding vulcanization for 10 min on a flat vulcanizer (QLB-50T) at 175 °C; finally, a second-stage vulcanization was performed in a blast drying oven at 200 °C for 4 h, and the FMC composite material was obtained after cooling. The specific formulations are shown in Table 1. The number is the content in the formulations. Figure 2 is the schematic diagram of the preparation process.

**Figure 1:**
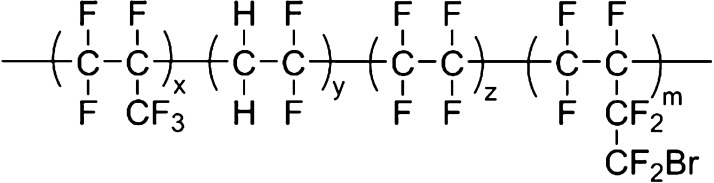
Molecular structure of fluororubbers.

**Figure 2:**
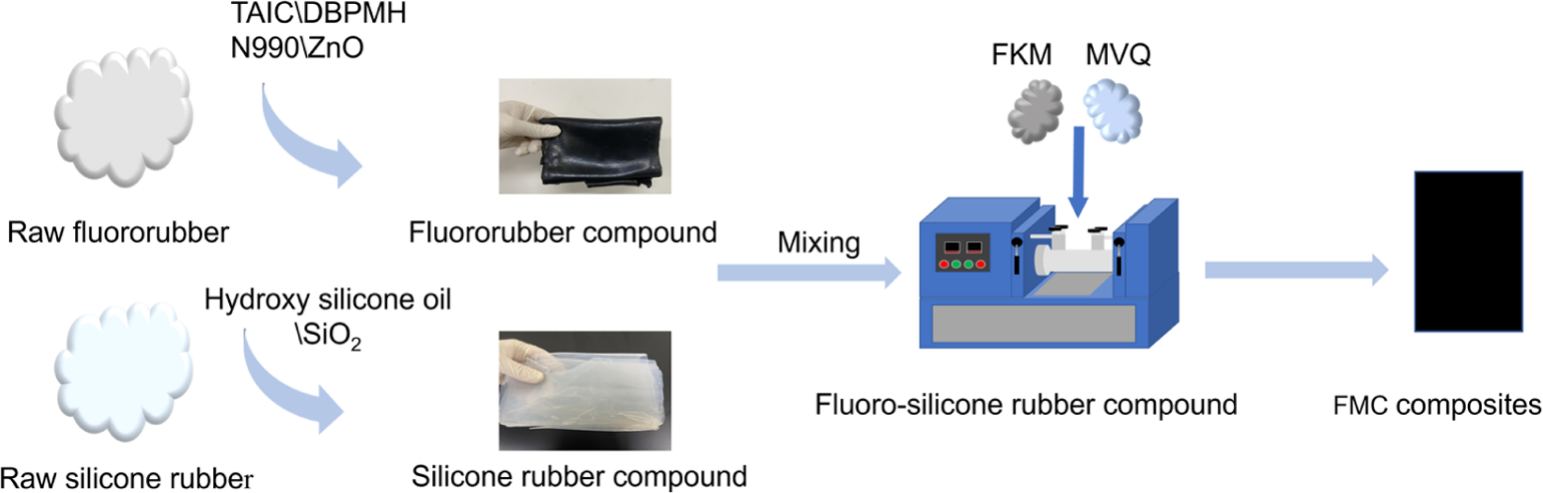
Schematic diagram of FMC composite process.

**Table 1:** Formulations of FMC Composites of Different FKM Types

sample	G506	G503	G203	MVQ	SiO_2_	N990	ZnO	hydroxy silicone oil	DBPMH	TAIC
unit	g	g	g	g	g	g	g	g	g	g
A1	90.00	0.00	0.00	10.00	4.00	28.00	4.50	0.20	1.45	3.15
A2	80.00	0.00	0.00	20.00	8.00	24.00	4.00	0.40	1.40	2.80
A3	70.00	0.00	0.00	30.00	12.00	20.00	3.50	0.60	1.35	2.45
B1	0.00	90.00	0.00	10.00	4.00	28.00	4.50	0.20	1.45	3.15
B2	0.00	80.00	0.00	20.00	8.00	24.00	4.00	0.40	1.40	2.80
B3	0.00	70.00	0.00	30.00	12.00	20.00	3.50	0.60	1.35	2.45
C1	0.00	0.00	90.00	10.00	4.00	28.00	4.50	0.20	1.45	3.15
C2	0.00	0.00	80.00	20.00	8.00	24.00	4.00	0.40	1.40	2.80
C3	0.00	0.00	70.00	30.00	12.00	20.00	3.50	0.60	1.35	2.45

### 2.3 Characterization and Testing

#### 2.3.1 Scanning Electron Microscopy Test

In order to study the microscopic morphology of FMC of this series of composite materials, the test was carried out by scanning electron microscopy (thermo scientific Apreo 2C) to observe the tensile section of the composite material and elemental analysis.

#### 2.3.2 Vulcanization Characteristic Test

In order to investigate the vulcanization performance of this series of FMC composite materials, a rotorless vulcanizer (2000E) was used to conduct the vulcanization test, the test temperature was 175 °C, and the test time was 10 min. The curing properties of the FMC composites are the incremental torque of the material (MH-ML), positive curing time (*T*_90_), and vulcanization rate index CRI.

#### 2.3.3 Compatibility Test

The compatibility of the composites was characterized by Fourier-transform infrared (FT-IR) spectroscopy, contact angle test, and swelling test. The FT-IR spectra of FKM, MVQ, and FMC were measured with a Fourier transform infrared spectrometer (INVENIO R), respectively, and the samples were tested in attenuated total reflection mode in the range of 4400–400 cm^–1^. The contact angle test uses a contact angle meter (C602) to characterize the wettability of the surface of the composite material, thereby evaluating the surface tension of the material. The surface energy of fluororubber and silicone rubber was determined by Owens two-liquid method, thereby indicating the compatibility of the composite material. First, we measure the samples FKM, MVQ, and FMC and cut them into contact angles of 2.5 × 2.5 cm. Then we measure with a contact angle meter at room temperature. The crosslink density was characterized by measuring the swelling index (SI) of the composites. The specific process is as follows: the weight is that the vulcanized rubber sample *W*_1_ is placed in a 250 mL conical flask, and the flask is filled with about 50 mL of xylene, sealed, and then soaked in xylene at 30 °C for 48 h until the equilibrium weight is reached. The swollen sample is quickly clamped onto dry, clean filter paper with tweezers. The solvent is gently wiped off from the swollen sample surface with filter paper. The mass *W*_2_ is noted exactly and corrected to 0.1 mg.^37^ The equation for calculating the SI is eq 1. The calculation formulae for surface energy are eqs 2 and 3
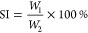
1where SI is the swelling index, *W*_1_ is the mass of FMC before swelling, and *W*_2_ is the mass of FMC after swelling
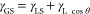
2where γ_GS_ is the saturated vapor of the liquid that reaches the surface tension of the equilibrium solid. γ_LS_ is the interfacial tension between the liquid and solid. γ_L_ is the surface tension of the liquid. θ is the contact angle of the liquid on the solid surface
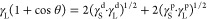
3where γ_L_^d^ is the surface tension of water, γ_L_^p^ is the surface tension of diiodomethane, and γ_S_^d^ is the surface tension of the polymer in water. γ_S_^p^ is the surface tension of the polymer in diiodomethane. Therefore, the surface energy of the polymer can be calculated.

#### 2.3.4 Oil Resistance Test

The oil resistance of the material is the key to high-temperature sealing. To test the oil resistance of the composites, a 25 × 50 mm standard sample was immersed in ASTM no. 1 standard oil at 150 °C for 70 h, and the change behavior of the material was observed. The equation for calculating the rate of change is as follows (eqs 4–6)
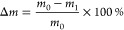
4where *m*_0_ is the initial mass of FMC and *m*_1_ is the mass of FMC after soaking
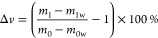
5where *m*_0_ is the mass of FMC in air before immersion, *m*_0w_ is the mass of FMC in distilled water before immersion, *m*_1_ is the mass of FMC in air after immersion, and *m*_1w_ is the mass of FMC in distilled water after immersion
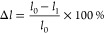
6where *l*_0_ is the size of FMC before immersion and *l*_1_ is the size of FMC after immersion.

#### 2.3.5 Thermal Performance

In order to analyze the stability, decomposition behavior, and performance maintenance of FMC composites at high temperature, thermogravimetric analysis was performed under a nitrogen atmosphere using a thermogravimetric analyzer (METTLER TOLEDO TGA/DSC 3+) with a heating rate of 10 °C/min; the test temperature is from room temperature to 800 °C, and it is evaluated by the change of mass decomposition at different temperatures. The composites were aged in a hot air oven at 200 °C for 48 h, and then the heat-aged samples were subjected to tensile testing to measure tensile strength and elongation at break. The retention rate was calculated by eq 7
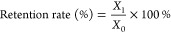
7where *X*_0_ is the performance value before aging and *X*_1_ is the performance value after aging.

#### 2.3.6 Mechanical Property Test

In order to test the physical and mechanical properties, the sample uses a universal testing machine (E43.504) for tensile test. The test speed is 500 mm/min. The hardness was measured using a type A shore hardness tester (GL02-HT-6510A). For the density of the sample using (FA2104J) measurements, first, we measure the composite material sample in the air, then we measure the sample in water, and finally, the density meter will read the density value of the sample. We test each sample three times to reduce the influence of error. At 200 °C, we select a compression rate of 25%, compress the sample under the fixture for 24 h, and calculate the permanent compression deformation rate of the sample. Physical and mechanical properties of the FMC composite are evaluated by the tensile strength, elongation at break, hardness, density, and permanent compressive deformation rate of the material.

#### 2.3.7 Dynamic Mechanical Analysis

In order to study the viscoelastic properties of FMC of this series of composite materials, a dynamic mechanical analyzer (Q800) was used in the test at −60 to 100 °C, the heating rate was 5 °C/min, the frequency was 1 Hz, and the amplitude was 20 μm, the test sample mode selection adopts a single cantilever fixture, and the viscoelastic properties of the material are represented by the glass-transition temperature tan δ and the storage modulus *E*′ of the material.

## 3 Results and Discussion

### 3.1 Microstructure Analysis

**Figure 3:**
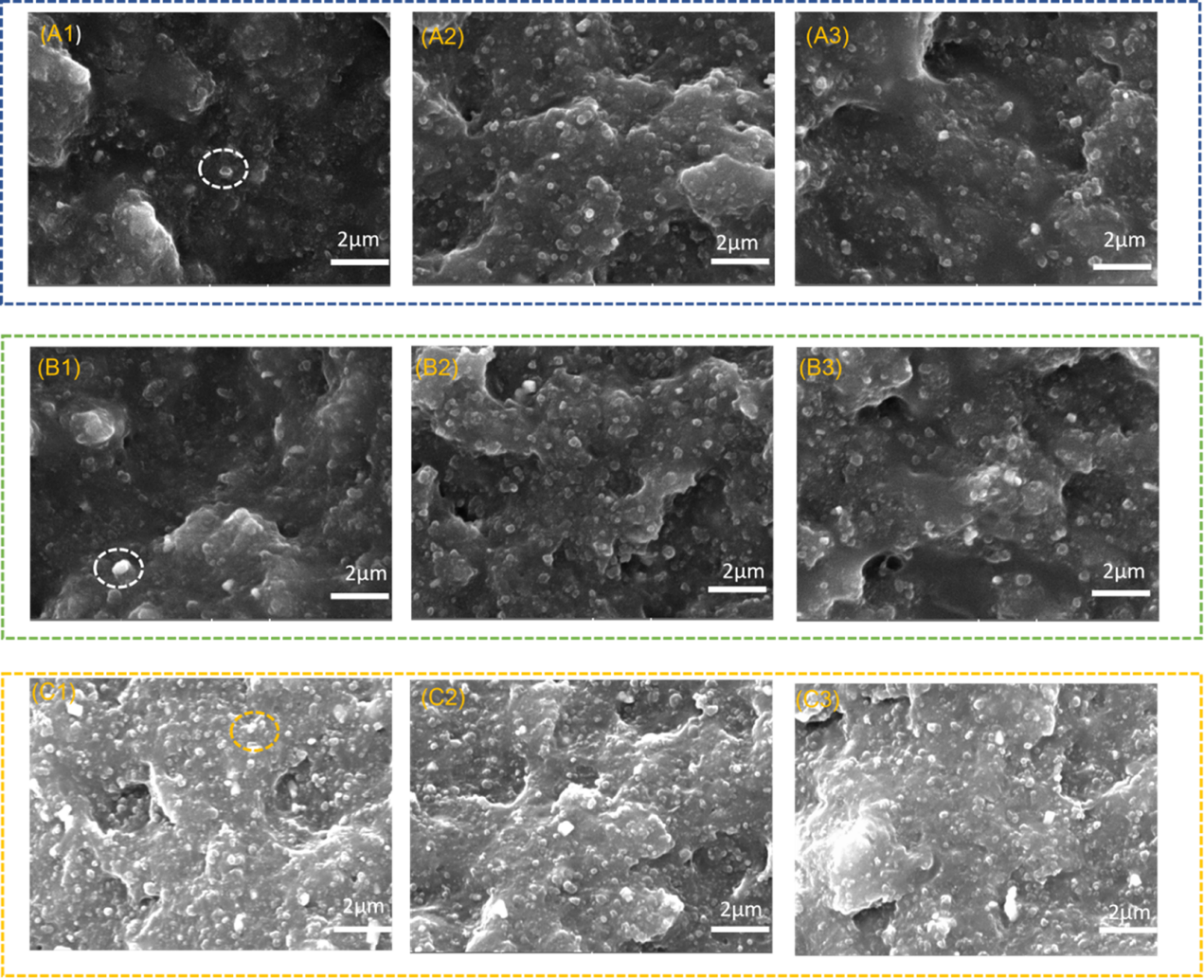
SEM images of FMC composites: (A) FKMG506, (B) FKM G503, and (C) FKM G203.

To research microstructures of the composites, a scanning electron microscope was used to observe the tensile sections of the composites. The result is shown in Figure 3; the raised part in the circle is also filled. As can be seen from Figure 3, the fillers are uniformly distributed on the nanometer scale. For composite materials, composite fillers are effective reinforcing materials. The images from left to right are the results of increasing MVQ additions. Due to the lower viscosity of MVQ, increasing the MVQ content results in more and more pores at the two-phase interface, which also reduces the compatibility of the composites. Specifically, the viscosity of MVQ is low, while the viscosity of FKM is high. When the two are combined, MVQ flows more easily and becomes the matrix phase, and FKM becomes the dispersed phase. According to thermodynamics, low-polarity MVQ and high-polarity FKM are thermodynamically incompatible systems, which cannot be avoided. However, the combination of too dispersed FKM and a small amount of MVQ has a smaller overall polarity gap than when MVQ increases, so the compatibility of the two can be alleviated. The two-phase bonding of the composite material is reduced to some extent. Through element detection, we can find that the overall element distribution is uniform, and the phase separation is very little, indicating that the mixed filler also plays a role in improving the compatibility. From top to bottom, the B-series compound rubber is smoother and smoother and is better combined than A and C, and the compound material at this time has better processability.

### 3.2 Vulcanization Properties

**Figure 4:**
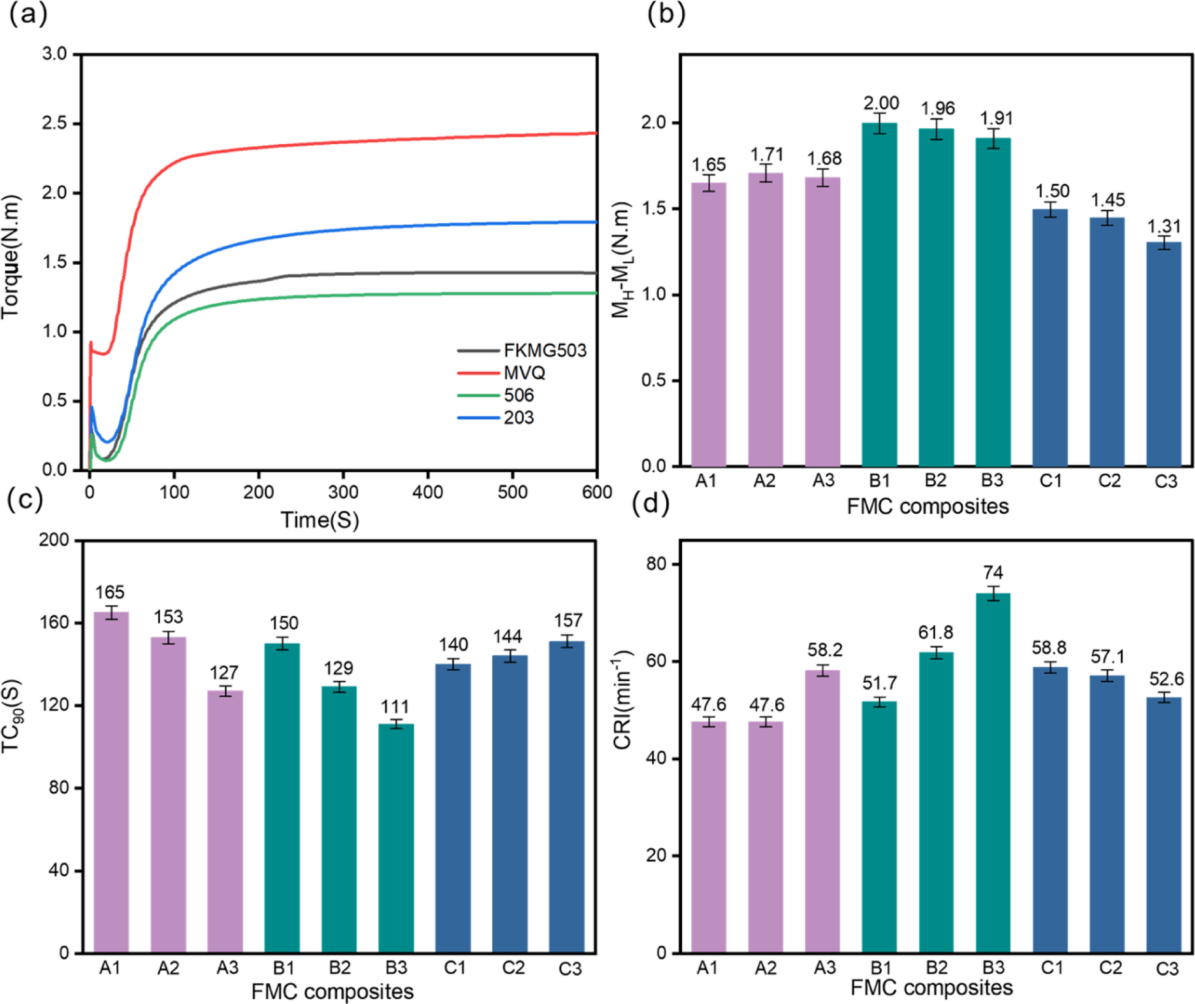
FMC composite vulcanization characteristic diagram: (a) vulcanization characteristic curves of FKM and MVQ. (b) Incremental torque MH-ML. (c) Positive curing time TC90. (d) Curing rate CRI.

The vulcanization performance of composite materials is an important factor to evaluate the comprehensive properties of composite materials. When under high temperature and pressure, silicone rubber will produce vinyl radicals and methylene radicals, and the two will crosslink. Fluororubber will produce bromine-containing radicals, and fluororubber will form a network structure in the presence of TAIC. Therefore, as long as it can be co-vulcanized, the composite will remain stable. Figure 4 shows the vulcanization properties of composites of different types and mixing ratios. Figure 4a are the curing curves of FKM and MVQ. It can be seen that the vulcanization rate of MVQ is better than that of FKM because the side chain of MVQ molecule has an ethylene double bond, which can react with peroxy radicals first in the same peroxy vulcanization system. During the vulcanizing process of FKM, it is necessary to perform free-radical activation on the vulcanization point monomer to generate double bonds and then perform free-radical reaction vulcanization.^38^Figure 4b is the incremental torque of the composite. It can be noted that the incremental torque of the B-series FMC composites is significantly better than that of the A-series and C-series, indicating that B has a higher crosslink density. Furthermore, with the increase of MVQ content, the silica content increased and the N990 content decreased, resulting in the inhibition in the vulcanization process and the decrease of its crosslinking density. Figure 4c is the positive curing time graph of the composites; the curing time *T*_90_ of the A-series and B-series composites decreased, and the curing time *T*_90_ of the C-series composites increased. Figure 4d is the curing rate of the composite. With the addition of MVQ, it can be seen that the cure rate CRI of the A-series and B-series composites increases, while the cure time CRI of the C-series decreases. The center is difficult to activate, hindering the vulcanization of the material. Therefore, for FMC composites, the vulcanization properties of the B-series are in a better state.

### 3.3 Compatibility

**Figure 5:**
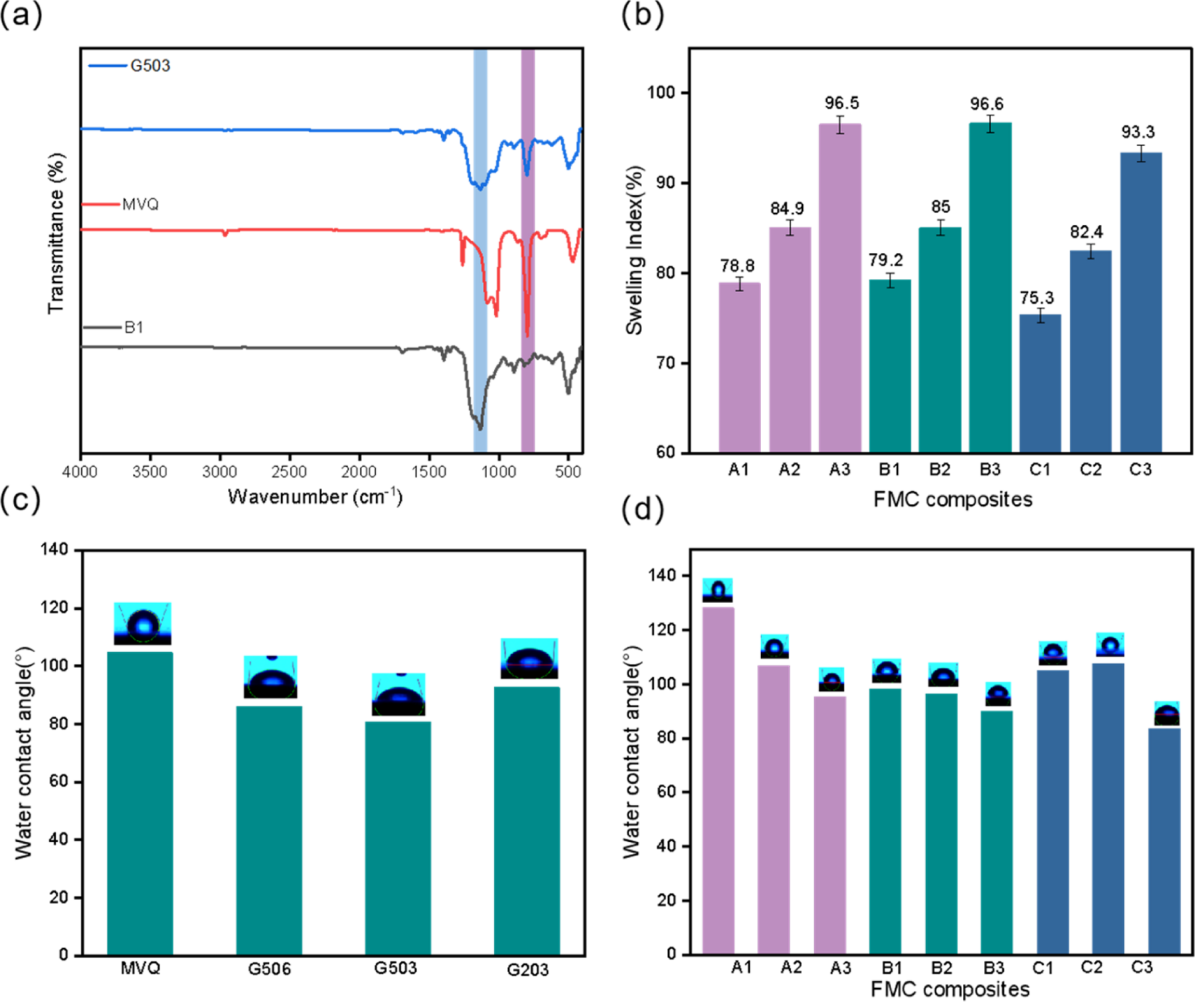
(a) Infrared spectra of FKM G503, MVQ, and composite B1 samples. (b) Histogram of SI of FMC composites. (c) Histogram of water contact plots of MVQ, G506, G503, and G203. (d) Histogram of water contact of FMC composites.

Compatibility is an important basis for determining the properties of composite materials. In this study, the changes in FT-IR, swelling property, and contact angle were used to reflect the compatibility changes of composite FMC. The infrared absorption peaks of FKM, MVQ, and FMC composites within 4000–400 cm^–1^ are shown in Figure 3a. The infrared spectrum of FKMG506 in Figure 5a corresponds to the characteristic absorption peaks of −CF_3_, −CF_2_, and −CF at 1394, 1131, and 891 cm^–1^, respectively. The characteristic absorption peaks of MVQ at 1295, 1081, and 795 cm^–1^ are assigned to the symmetrical −Si–C symmetric peaks, −Si–O peaks, and −Si–C stretch peak, respectively. The above characteristic peaks appear in FMC materials, but their characteristic partial shift of the peak occurred, which may be due to the small polarity of MVQ and the bonding effect with fumed silica. The content of FKM itself is low after a small amount is introduced into FKM, which leads to the shift of the characteristic peak. On the whole, FKM and MVQ in FMC materials have a certain combination and have a certain compatibility. Rubber materials expand under the action of solvents, and there is a certain compatibility. Rubber materials will swell under the action of solvents, so the crosslinking density of rubber can be indirectly reflected by testing the swelling behavior of composite FMC under medium conditions. The smaller the SI under the same constant temperature solvent condition, the higher the crosslinking density of FMC composites and the better the compatibility of high crosslinking density composites.^37^Figure 5b shows that the introduction of MVQ reduces the crosslinking density of the composites. During the mixing process, when a small amount of MVQ is introduced, the FKM phase is still dominant, and the MVQ vulcanization rate is fast and has little effect on the vulcanization of the composite material. The influence on the composite material is intensified. With the changes of the content of silica and N990 in the system, the increase of silica hinders vulcanization, reduces the three-dimensional network structure, and increases the expansion index. In addition, the SI can also reflect the tolerance of the composite material to organic solvents, and the improvement of the compatibility helps the composite material to have good solvent resistance. The surface wettability of composite materials can reflect the surface tension of materials and also reflect the structural properties of materials. The surface wettability of FKM, MVQ, and FMC was experimentally investigated. Through comparative analysis, it is found that the change of wettability reflects the compatibility of FMC of composites to a certain extent. Figure 5c shows the water contact angles of MVQ and three FKMs. The water contact angle of MVQ is 107°, so it shows hydrophobicity. However, G503 is 88.3°. In theory, due to the polarity gap between two rubbers, the water contact angle of silicone rubber is lower than that of fluororubber. However, due to the introduction of hydrophilic carbon black N990, the surface roughness changed and the contact angle decreased. Then the surface energy of the two was calculated through the contact angle data. The surface energy of MVQ is 56.14 mN/m, that of G506 is 34.52 mN/m, that of G503 is 30.09 mN/m, and that of G203 is 42.56 mN/m. It can be seen that the surface energy of MVQ is significantly greater than that of FKM, so the two are not easy to blend due to the difference in surface energy. Figure 5d shows the water contact angle of the composite material. Compared with the single MVQ or FKM, the water contact angle of the composite material is increased. Guo^39^ studied the effect of mixed fillers and pointed out that the mixed introduction of chemical fillers can effectively increase the loading of fillers, resulting in higher mixed filler polymers and filler networks. The results show that the composites formed by MVQ and FKM with the help of N990 filler and silica have the effects on reducing the surface energy, the contact angle, and the surface tension and improving the compatibility of FMC composites.

### 3.4 Oil Resistance

The oil resistance of the material limits the application of composites, which is currently the key to high-temperature sealing. To study the oil resistance of FMC composites, the samples were immersed in ASTM no. 1 standard oil at a temperature of 150 °C for 70 h. The results in Table 2 showed that the composites performed well in ASTM no. 1 low-expansion oil. Among them, the mass change showed a small change, A1 to A3 increased from 0.11 to 0.77%, B1 to B3 increased from 0.11 to 0.94%, C1 to C3 increased from 0.27 to 1.06%, and the volume change has the same change rule as the size. This shows that with the addition of MVQ, the change in the amount of silica and N990 in the composite system will barely affect its excellent oil resistance. In this system, FKM is still the majority. Under the conditions of the standard oil medium, most of the surface contacting standard oil is the FKM phase in the composite material, which is attributed to the introduction of F atoms in FKM, making it have good oil resistance.

**Table 2:** Change of FMC Composites under Standard Oil Conditions

equation	mass change rate (%)	volume change rate (%)	length change rate (%)	width change rate (%)
A1	0.11	0.22	0.02	0.08
A2	0.34	0.57	0.21	0.89
A3	0.77	1.70	0.46	0.60
B1	0.11	0.36	0.20	0.16
B2	0.54	1.29	0.20	0.18
B3	0.94	1.89	0.37	0.61
C1	0.27	0.77	0.34	0.83
C2	0.75	1.83	0.22	0.53
C3	1.06	2.27	0.26	0.35

### 3.5 Thermal Properties

**Figure 6:**
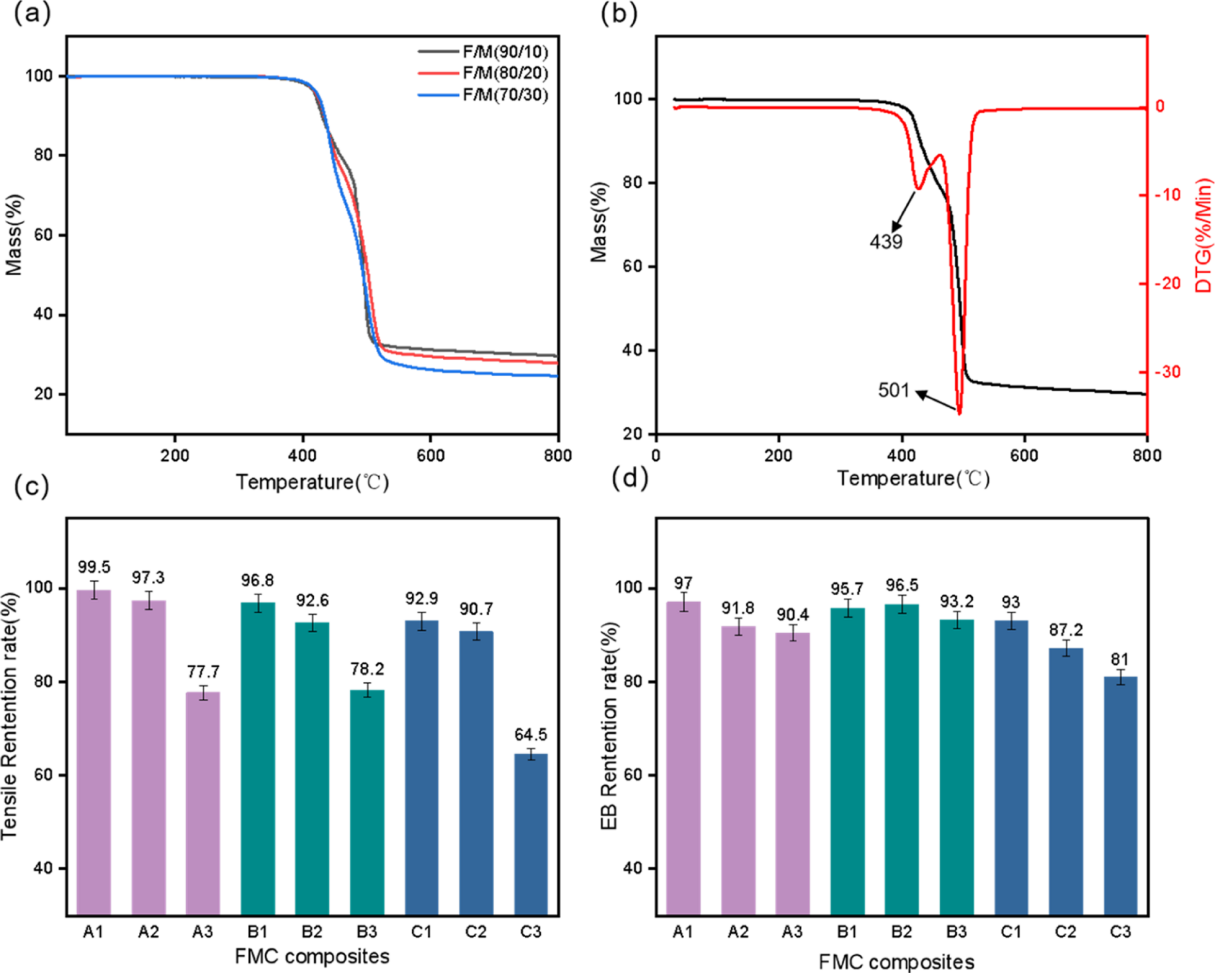
(a) TG curve of B-series composites. (b) TG-DTG curve of composite B1 sample. (c) Histogram of tensile strength retention rate. (d) Histogram of elongation at break retention rate.

In order to study the high-temperature stability of FMC composites, two methods, thermogravimetric analysis and high-temperature aging, were used. The results are shown in Figure 6. In Figure 6a, it can be seen that MVQ prolongs the decomposition end temperature, while B1 begins to decompose at 410 °C, B2 begins to decompose at 422 °C, and B3 begins to decompose at 429 °C. The remaining parts are SiO_2_, ZnO, and part of the FKM, and the analysis curve can get the corresponding proportion of the composite material, which is consistent with the actual condition. The addition of MVQ will cause the composite rubber to begin to degrade with temperature hysteresis, indicating that the heat resistance of silicone rubber is better than that of fluororubber to a certain extent, and silicone rubber can effectively improve the thermal stability of composite rubber. As can be seen from the B1 thermogravimetry–derivative thermogravimetry (TG-DTG) map in Figure 6b, this compound has two temperature decreasing trends. We analyze the degradation behavior of fluororubber and silicone rubber due to the pyrolysis of the C–C main chain in FKM, so a small part of FKM is degraded at 400–431 °C, and most of it is degraded at 431–500 °C. The residues are N990, TAIC, and ZnO; the proportions of the whole are 21.43, 2.50, and 3.57%, respectively. Because of the pyrolysis of the Si–O–Si main chain in MVQ, it begins to degrade at 400 °C, and the final remaining substance is SiO_2_, accounting for 27.26% of the whole.^40^ The first stage between 410 and 439 °C is the decomposition of a small fraction of the fluororubber in the composite, and the second stage occurs between 439 and 500 °C. During this period, most of the fluororubber degraded and the silicone rubber also degraded. At the same time, we found that the type of fluororubber has little effect on the thermal stability of the composite. The aging curves for A, B, and C show the same trend, and the curves are very close at high temperature. In Figure 6c, it can be found that after 48 h at 200 °C, the tensile strength of the composite is hardly affected at the blend ratio of 90/10 and 80/20. A1 is 99.5%; B1 reaches 96.8%. At a mixing ratio of 70/30, the proportion of MVQ in FMC increased, while the increase in MVQ could not be fully covered by FKM. During thermal aging, structural changes occur first, which tend to degrade performance. The retention of elongation at break can be expressed as Figure 6d.Under high temperature for a long time, the composite material still maintains excellent tensile properties, among which A1 is 97.0%, B1 is 95.7%, and C1 is 93.0%, so the composite material A1 has the best anti-aging ability, but on the whole, G503 has a more stable performance, indicating that at high temperature, the degree of structural change is minimal. Therefore, B-series FMC composites are more stable at high temperature and can be used in high-temperature media for a long time.

### 3.6 Mechanical Properties

**Figure 7:**
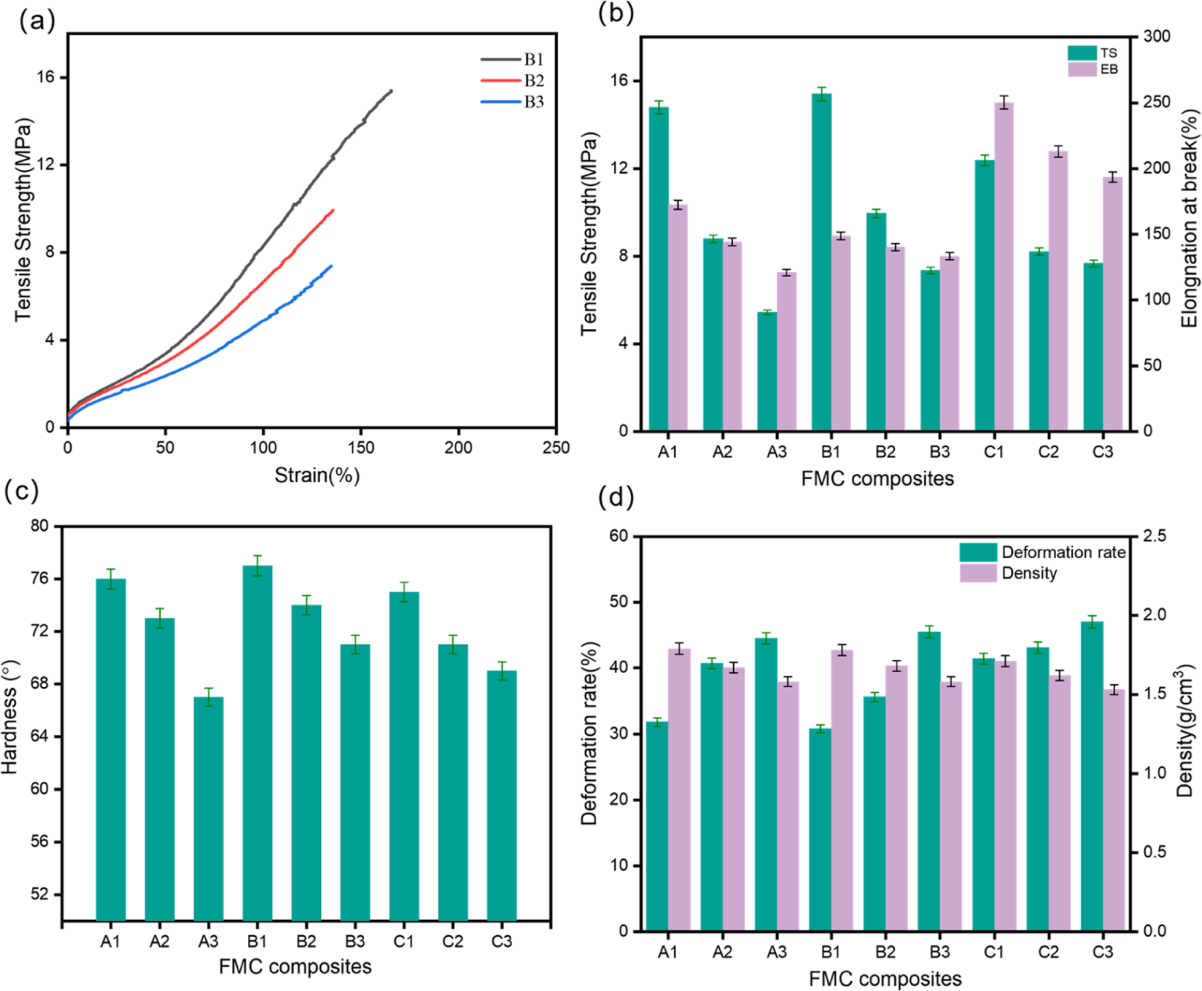
Mechanical properties of FMC composites: (a) B-series stress–strain curve. (b) Histogram of tensile strength and elongation. (c) Histogram of hardness. (d) Histogram of compression ratio and density.

Mechanical properties are important criteria for rubber application. The effects of fluororubber varieties and blending ratios on the mechanical properties of FMC composites were explored. The tensile properties, compressibility, density, and hardness of the materials were measured, as shown in Figure 7. Figure 7a is the stress–strain curve of composite material B. With the gradual addition of MVQ, the silica content increases, the curve increases in a short time, the rigidity of the material increases, and the mechanical properties of the composite material show a decreasing trend. The mechanical properties of silicone rubber itself are worse than those of fluororubber, and the two are thermodynamically incompatible systems. The existence of MVQ will lead to the decline of FKM performance, so the composites with a small amount of MVQ have smaller interfacial tension and better material compatibility. The multi-stacking increases the surface tension of the interface, which leads to the incompatibility of the materials. In Figure 7b, the maximum tensile strength of B1 is 15.4 MPa, which indicates that the FMC composite has the best bonding force and better mechanical properties. The maximum elongation of C1 is 250% due to the low crosslink density of the composite, which is due to the low stress concentration during testing. Figure 7c presents the relationship between the blending ratio of FKM/MVQ and the hardness of the material. B3 is higher than A3 and C3. The increase in the amount of addition leads to a continuous phase of the silicone rubber in the composite material ratio of 70/30, while the silicone rubber is the dispersed phase, leading to the hardness of the composite material. In Figure 7d, it is found that the introduction of MVQ will increase the compressibility of the material, which shows an upward trend. Among them, A1 is 31.8% and B1 is 30.8%, which are significantly lower than C1’s 41.4%. The results show that the properties of fluororubbers of the same grade of A and B are similar when the blending ratio is 90/10, while fluororubber C is deviated. This may indicate that the fluororubber of the composite material is a continuous phase at 90/10, while the silicone rubber is a dispersed phase. Further, when the blending ratio is 80/20, it was significantly found that B2 is 35.6%, which is significantly lower than A2’s 40.7% and C2’s 43.1%, indicating that the increase in MVQ has less effect on the performance of B-series. MVQ is lower than FKM because of its density. Therefore, the addition of MVQ will gradually reduce the density of the composite material, but under the same blending ratio, fluororubbers A and B are obviously better than fluororubber C. The experimental results show that the same grade of fluororubber has a lower Mooney viscosity. The mechanical properties of the material are better than that of the A-series, while the B- and C-series have the same Mooney viscosity. When B is compounded with the silicone rubber, the comprehensive mechanical properties are better, and overall, it has high strength. The three fluororubbers used in this article are all peroxide fluororubbers, and the purpose of selection is to facilitate the co-vulcanization of composite materials in the peroxide vulcanization system. The molecular formulas of the three fluororubbers are all listed in Figure 1, and they are all composed of three monomers and vulcanization point monomers. Among them, the content of vulcanization point monomer in G506 and G503 is higher than that of G203, and the overall molecular weight and fluorine content are higher. Compared with 506, 503 has a lower Mooney viscosity and is easier to process, so G503 has better comprehensive performance.

### 3.7 Dynamic Mechanical Analysis

**Figure 8:**
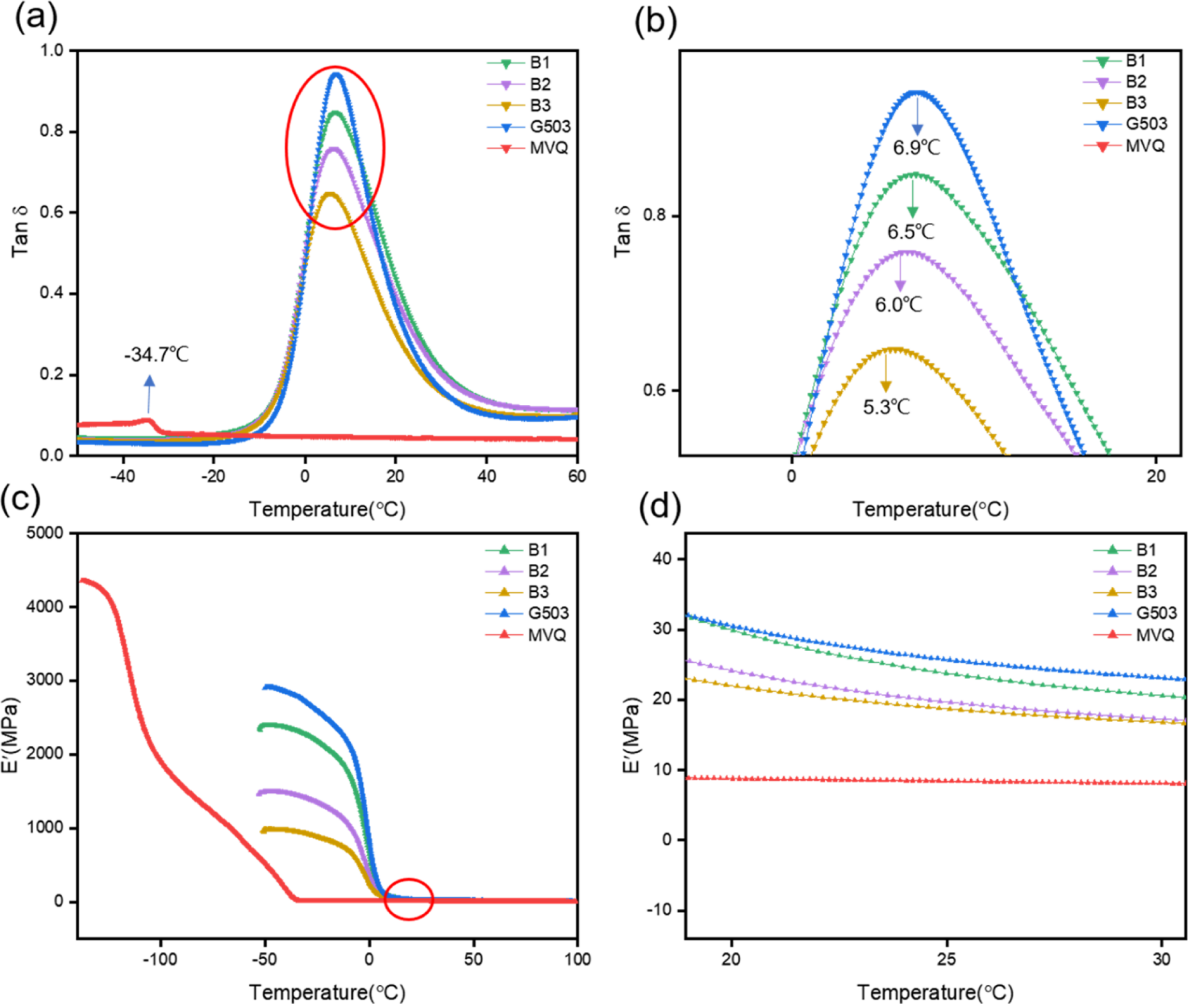
DMA curves of FMC composites: (a) tan δ curves of FKM, MVQ, and B-series composites. (b) Enlarged part of (a). (c) Storage modulus (*E*) curves of FKM, MVQ, and B-series composites. (d) Enlarged part of (c).

The dynamic mechanical analysis (DMA) of the composites is shown in Figure 8. As shown in Figure 8a, the *T*_g_ of MVQ is −34.7 °C and that of FKM is 6.9 °C. The relationship of the composite material *T*_g_ is B1 > B2 > B3 > G503. The composite shows a weak peak around −30 °C, which is the low content of MVQ and high content of FKM, just corresponding to the peaks of MVQ and FKM in the composite material. Figure 8b shows that the glass-transition temperature *T*_g_ of the composites shifts to the left after the introduction of MVQ, indicating that the effect of the composites is improved after the introduction of MVQ. Due to the difference in polarity between the two composite materials, it can be concluded from Figure 8c that MVQ has a faster response, and the magnitude relationship of its *E* at the same temperature is G503 > B1 > B2 > B3 > MVQ, indicating that FKM is better than FKM. MVQ has more rigidity. After the energy consumption process reaches the *T*_g_ temperature, the storage modulus *E*′ shows a constant trend, indicating that the energy has been reduced to the minimum. Generally, composite material B has good damping performance.

### 3.8 Formation Mechanism of FMC Composites

**Figure 9:**
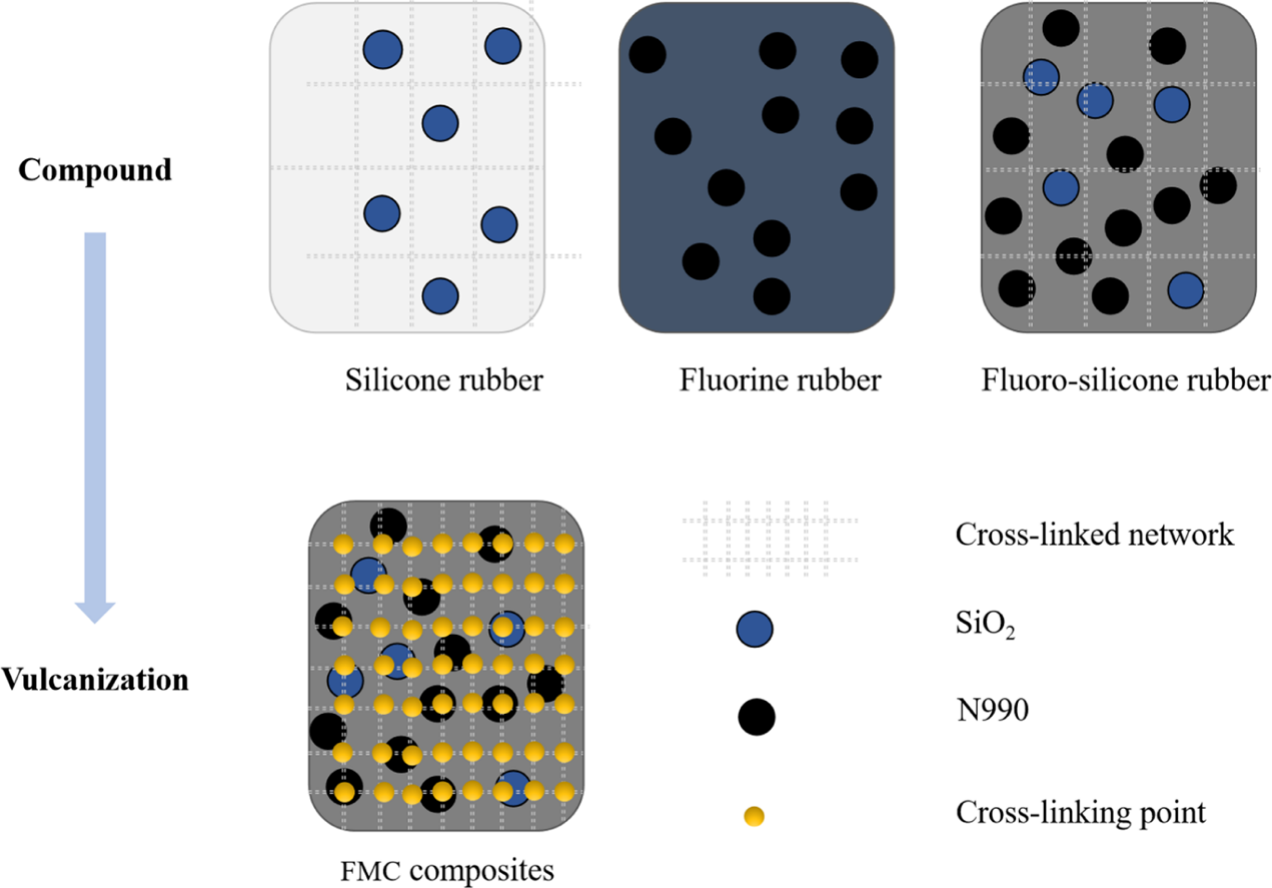
Schematic of the formation mechanism of FMC composites.

In the formation mechanism of the composite material, due to the synergy between its fillers, SiO_2_ is first added to the silicone rubber, and N990 is then added to the fluororubber. In this way, the substrate and the filler can be fully combined, and the composite phase separation of the two can be reduced to a certain extent. Second, SiO_2_ and N990 in the fluorine/silicone premix prepared by compounding are evenly distributed, and this method for mixing can make the blending of the composite material sufficient. Due to the existence of intermolecular forces, there is also a certain interaction between the two phases in the premix, which potentially increases the crosslinked network during the vulcanization process. Finally, when vulcanized at high temperature and high pressure and induced with peroxide vulcanizing agent, the crosslinking points gradually increased; that is, the number of yellow points in Figure 9 increased, along with the gradual increase of the vulcanization sites of the system. Forming a polymer three-dimensional network composite material, the matrix fluororubber as the main part, MVQ as the added part can be well compatible, and the synergy of the filler can give the composite material excellent mechanical, thermal, and medium resistance and aging resistance.

## 4 Conclusions

In this study, FMC composites were efficiently prepared by mechanical blending method. The composites have good sulfuration behavior. FT-IR, mechanical properties, and contact angle test thermodynamics show the improved compatibility of FMC composites, reducing the phase separation between FKM/MVQ, and then the interface contact is better. Silica and N990 used as fillers are synergistic, which facilitates the combination of FKM and MVQ and improves the properties of the composites. The study found that the optimal blending ratio of FMC is 90/10, and the strength of the composite material can reach 15.4 MPa at this time, which has an obvious high strength. Comparing three different fluororubbers, it is found that the G503 series composites have the best comprehensive performance under the same conditions. At the same time, experimental analysis data show that FMC composite material has excellent aging resistance, oil resistance, medium resistance, and high/low-temperature performance, which can effectively broaden the scope of use. Compared with expensive fluorosilicone rubber, FMC material has a lower cost and an excellent economic value, also confirming the feasibility of this study.

## Supporting Information Available

Prepared composites and various characterizations and testings (PDF)
