# Supplementary material for: Preparation of High-Strength and Excellent Compatibility Fluorine/Silicone Rubber Composites under the Synergistic Effect of Fillers
Source: ACS Omega. 2023 Jan 17;8(4):3905–16. doi: 10.1021/acsomega.2c06489 (PMC9893473; doi:10.1021/acsomega.2c06489)
Supplement: Supplementary file 1 — ao2c06489_si_001.pdf [file ao2c06489_si_001.pdf]

Supporting Information

Preparation of high strength and excellent compatibility fluorine/silicone rubber composites under the synergistic effect of fillers

RongPeng Zhao[a,b], ZhiGang Yin[a,b], Wei Zou[a,b]*, Hu Yang[a,b], Jie Yan[a,b], WenJiang Zheng[a,b], and Hui Li[c]

[a]*School of Chemical Engineering, Sichuan University of Science & Engineering, Zigong, 643000, China.*

[b]*Organic Fluorine Material Key Laboratory of Sichuan Province.Zigong, 643000, China.*

[c]*Zhonghao Chenguang Chemical Research Institute, Zigong 643201, China.*

E-mail address: chzouwei@suse.edu.cn

Figure of Contents

Figure S1. Sample diagram of the prepared composite material. S3-S4

Figure S2. SEM and mapping for B1 material, (a)SEM of B1 material,(b)C elements,(c)F elements,(d) O elements, (e)Si elements. S4-S5

Figure S3. TG curve of MVQ and G503. S6

Figure S4. TG-DTG curve of MVQ andG503. S6

Figure S5. Stress-strain curve of G503 and FKM. S6-S7

Figure S6. DSC curve of G503 and composite material. S7-S8

Table of Contents

Table S1. Water contact angle, diiodomethane contact angle, and surface free energy data. S8

Supporting figures

Preparation process sample diagram

In order to make it easier for readers to understand the preparation process of fluorine/silicon composite rubber materials, we provide a schematic diagram of the sample preparation process, as shown in Figure S1. Firstly, the fluororubber raw rubber and various additives are fully stirred in the internal mixer, and then quickly transferred to the open rubber mixer for secondary processing. At this stage, vulcanizing agents will be added to carry out rubber processing operations (cutter, turning Glue, Botong, triangular bag) to ensure that the fluororubber compound meets the requirements. The processing process of raw silicone rubber is the same as that of fluororubber. Next, roll fluorine rubber raw rubber on the open mill, and add silicone rubber raw rubber several times in a small amount. This step needs to adjust the roller distance to about 1mm, and then adjust the roller distance to 2mm when discharging, so as to facilitate the next step of vulcanization. The composite material needs to be parked for more than 4 hours before vulcanization.

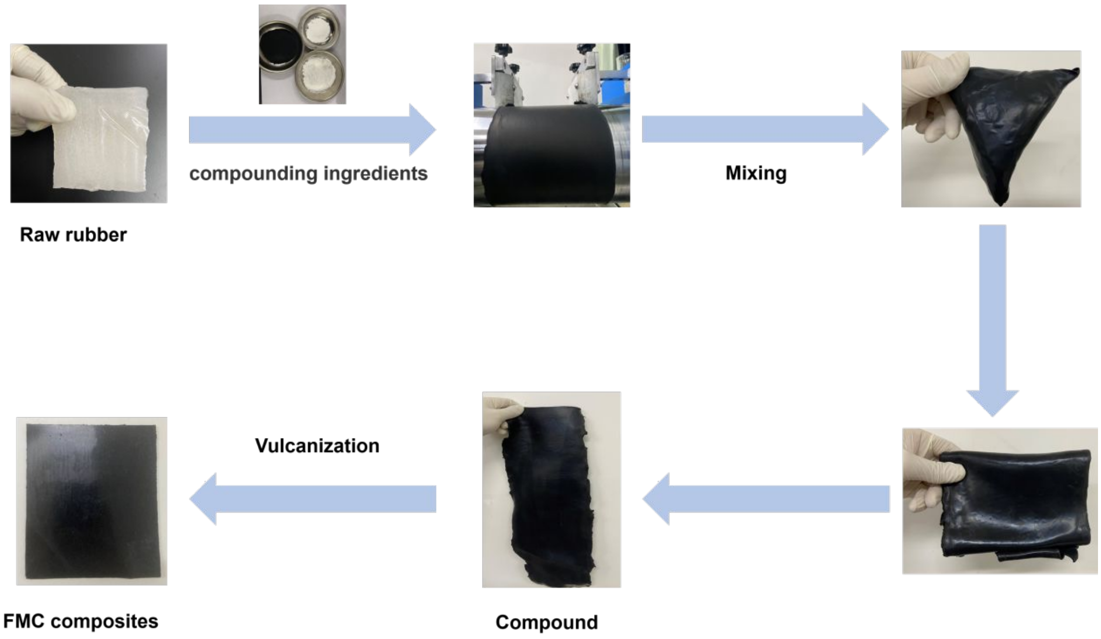

Figure S1. Sample diagram of the prepared composite material.

Energy Dispersive Spectrometer(EDS)

The elemental analysis of characteristic sample B1 is shown in Figure S2 below. The four elements of C, F, Si, and O are uniformly distributed as a whole. It can be seen that the two phases of FKM and MVQ in the composite material are fully combined, and there is no obvious phase separation. indicating that the mixed filler also plays a role and improves the compatibility.

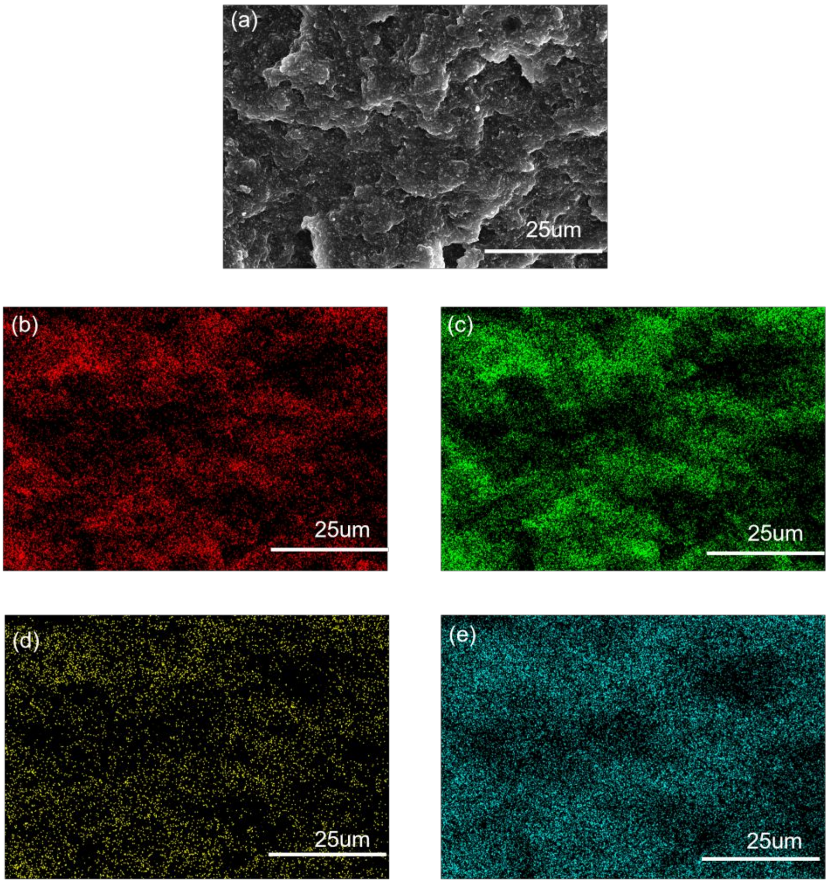

Figure S2. SEM and mapping for B1 material, (a)SEM of B1 material,(b)C elements,(c)F elements,(d) O elements, (e)Si elements.

TG-DTG analysis

It can be seen from Figure S3 and S4 that due to the pyrolysis of the C-C main chain in FKM, a small part of it is degraded at 400-431°C, and most of it is degraded at 431-500°C,This corresponds exactly to the two degradation trends in S4(a). and the residues are N99O, TAIC, and ZnO. The proportions of the whole are respectively for: 21.43%, 2.50%, 3.57%. In Figure S4 (b), due to the pyrolysis of the Si-O-Si main chain in MVQ, it begins to degrade at 400 °C, and the final remaining substance is SiO_2, accounting for 27.26% of the whole.

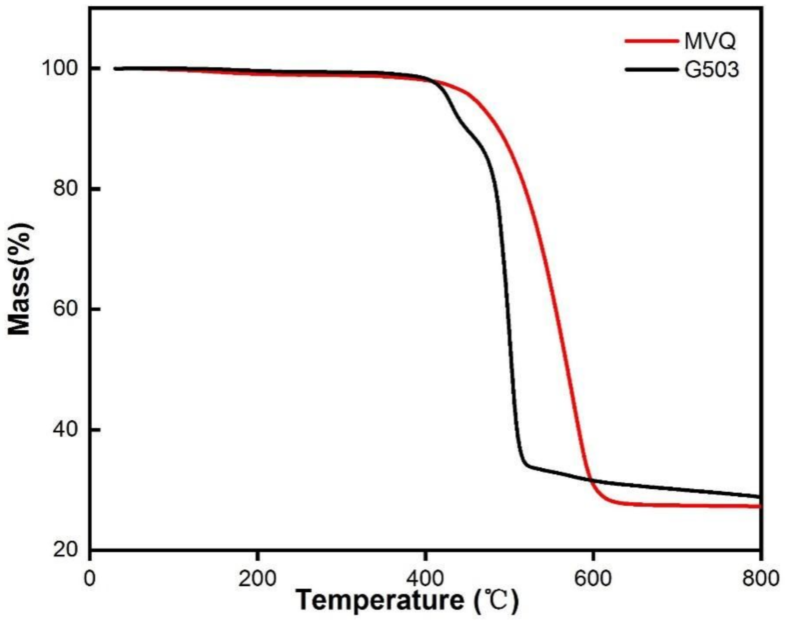

Figure S3. TG curve of MVQ and G503

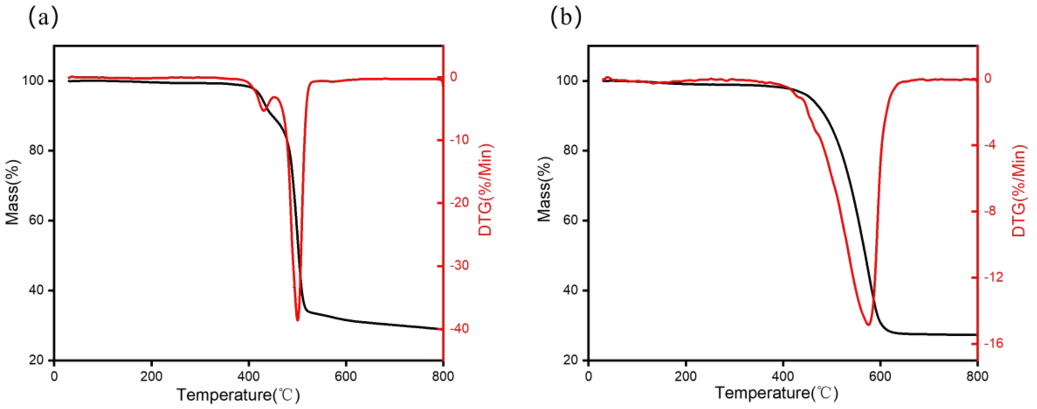

Figure S4. TG-DTG curve of MVQ and G503, （a）TG-DTG curve of G503, (b)TG-DTG curve of MVQ

Mechanical property test

The mechanical properties of silicone rubber itself are worse than those of fluororubber ,From Figure S5, it can be seen that the mechanical strength of silicone rubber is significantly lower than that of fluororubber, and the elongation at break is higher than that of fluororubber. The combination of the

two can improve the processing performance of fluororubber to a certain extent.

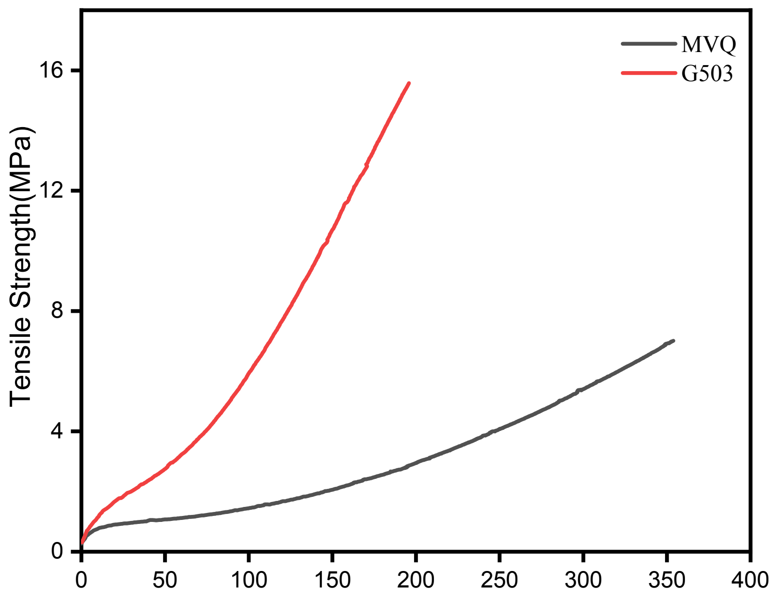

Figure S5. stress-strain curve of G503 and FKM

DSC analysis

We supplemented the DSC test of the composite material, and found that the glass transition temperature of G503 is -2.8°C. With the addition of MVQ, the glass transition temperature tends to decrease, and B3 can reach -4.5°C, The low temperature performance of the composite material can improve , so the compatibility has also been improved to a certain extent.

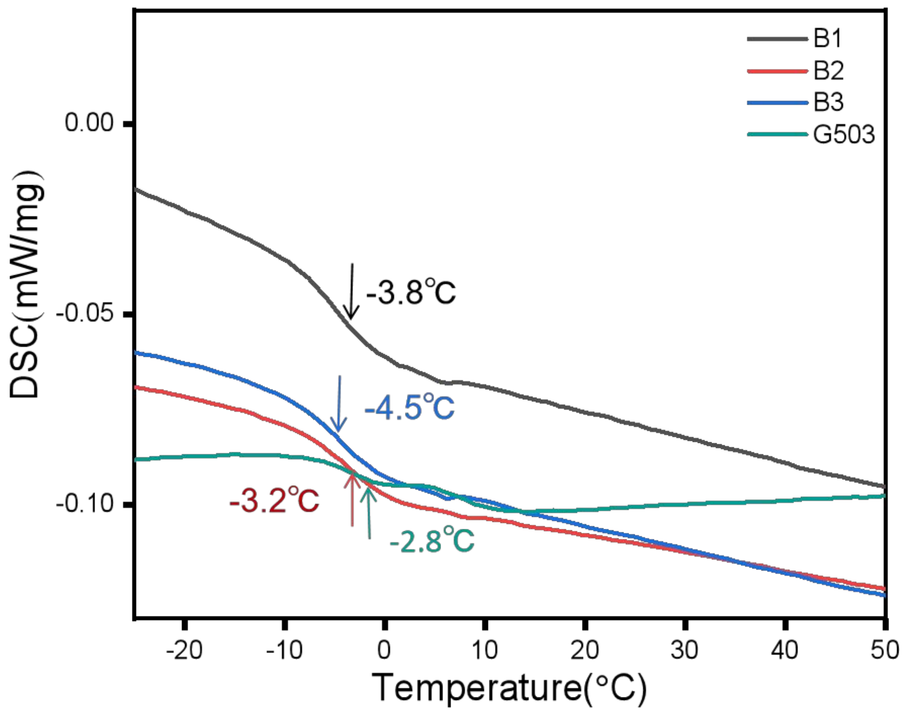

DSC curve of G503 and composite material.

Surface energy analysis

Table S1. Water contact angle, diiodomethane contact angle, and surface free energy data.

sample	Water contact angel (°)	Diiodomethane contact angel(°)	Surface free energy (mN/m)
MVQ	107.5	83.04	56.14
G506	76.39	85.71	34.52
G503	88.44	90.00	30.09
G203	89.17	81.84	42.56

Surface energy of fluororubber and silicone rubber are as table 1. We calculated the surface energy of the two based on the contact angle data. The surface energy of MVQ is 56.14 mN/m, that of G506 is 34.52 mN/m, that of G503 is 30.09 mN/m, and that of G203 is 42.56 mN/m. It can be seen that the surface energy of MVQ is Obviously larger than FKM, so the two will not be easy to blend due to the difference in surface energy.
